# Grisel’s syndrome, a rare cause of anomalous head posture in children: a case report

**DOI:** 10.1186/s12886-016-0197-1

**Published:** 2016-03-01

**Authors:** Davide Allegrini, Alessandro Autelitano, Elisabetta Nocerino, Paolo Fogagnolo, Stefano De Cillà, Luca Rossetti

**Affiliations:** Eye Clinic, San Paolo Hospital, University of Milan, Via di Rudini’ 8, 20142 Milan, Italy; Unit of Ophthalmology, Melegnano Hospital, Vizzolo Predabissi, Milan, Italy; Unit of Radiology, San Paolo Hospital, University of Milan, Milan, Italy; Unit of Ophthalmology, Maggiore della Carità Hospital, Novara, Italy

**Keywords:** Anomalous head posture, Grisel’s syndrome, Non-traumatic atlanto-axial subluxation, Ocular motility

## Abstract

**Background:**

Anomalous head posture (AHP) or torticollis is a relatively common condition in children. Torticollis is not a diagnosis, but it is a sign of underlying disease. Grisel’s syndrome (GS) is a rare condition of uncertain etiology characterized by a nontraumatic atlanto-axial subluxation (AAS), secondary to an infection in the head and neck region. It has not been considered, in ophthalmological papers, as a possible cause of AHP.

**Case presentation:**

A case of AAS secondary to an otitis media is studied. The children showed neck pain, head tilt, and reduction in neck mobility. The patient had complete remission with antibiotic and anti-inflammatory therapy and muscle relaxants. Signs of GS should always be taken into account during ophthalmological examination (recent history of upper airway infections and/or head and neck surgeries associated to a new onset of sudden, painful AHP with normal ocular exam). In such cases it is necessary to require quick execution of radiological examinations (computer tomography and/or nuclear magnetic resonance), which are essential to confirm the diagnosis.

**Conclusion:**

GS is a multidisciplinary disease. We underline the importance of an accurate orthoptic and ophthalmological examination. Indeed, early detection and diagnosis are fundamental to achieve proper management, avoid neurological complications and lead to a good prognosis.

## Background

Anomalous head posture (AHP) or torticollis is a relatively common condition in children, with an estimated prevalence of 5,6 % in general ophthalmological practice. The prevalence in pediatric practice is 3,19 [1]. Ballock and Song retrospectively analyzed 288 patients seen in a tertiary care pediatric orthopedic facility for an AHP. 18.4 % (53 patients) of these children had a nonmuscular etiology for their torticollis. Of these 53 patients, Kippel-Feil anomalies were present in 16 (30 %), and neurologic disorders were present in 27 (51 %). These neurologic conditions included ocular disorders in 12 cases (23 %) [2].

Torticollis is not a diagnosis, but a sign of an underlying disease. Assessment of AHP might be biased by physician’s training, experience and patient’s clinical presentation. Clinicians will have different points of view on AHP based on their specialization and clinical practice. In ophthalmological literature the following have been described as common causes of AHP: congenital or acquired muscular disease involving the muscles of the neck; congenital or acquired disease of the spinal column; abnormal neural inputs, also included visual pathway, oculomotor nerves and the vestibular apparatus [1–3].

Further causes of ocular AHP are: essential infantile esotropia, incomitant strabismus (superior oblique muscle deficit or palsy, lateral rectus muscle palsy, Duane syndrome, Brown syndrome, dissociate vertical deviation, A- and V-patterns, monocular elevation deficiency, inferior oblique palsy, third nerve palsy), nystagmus, refractive errors, neuro ophthalmological disorders and a group of pathologies that prevent the foveal fixation like palpebral ptosis [1, 4, 5]. In ophthalmological papers, Grisel’s syndrome (GS), which is a rare cause of AHP and it consists of a non-traumatic atlanto-axial subluxation (AAS), has not been previously considered as a possible cause of AHP. It is equally frequent in both males and females, occurring almost exclusively in children and young adults: 68 % of reported patients are under 12 years of age and 90 % are under 12 years of age [6]. However, this condition has also been described in patients ranging from infancy to the seventh decade of life [7].

GS has been associated with head and neck infection [8–11] such as pharyngitis, adenotonsillitis, tonsillar abscess, cervical abscess and otitis media. It has also been observed after numerous otolaryngological procedures such as tonsillectomy, adenoidectomy, mastoidectomy, choanal atresia repair, and excision of neck tumors [11–15]. GS is a multidisciplinary disease, the correct diagnosis is challenging and requires a strong clinical suspicion and appropriate radiographic imaging.

We report a case of GS to underline the importance of early diagnosis for an appropriate and successful treatment in ophthalmological daily practice. This is the first description about ophthalmological evaluation in a case of GS.

## Case presentation

**Fig. 1:**
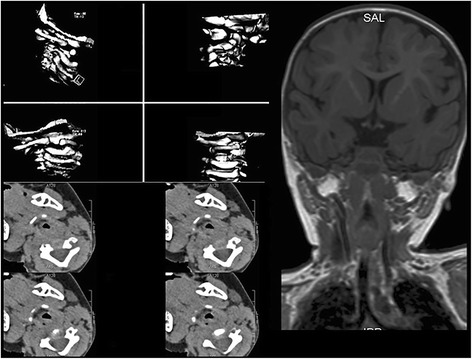
3D-CT reconstruction, axial and anterior-posterior NMR scan showing AAS and the typical head tilt

A child, 1 year old, male, was referred to our outpatient clinic of pediatric ophthalmology for an AHP. He had been examined at the Emergency Department 3 days earlier, where he was diagnosed with otitis media, pharyngitis with fever and AHP. The neurological examination didn’t reveal any abnormality. Therefore the otolaryngologist prescribed oral antibiotics and required an ophthalmological evaluation to assess the AHP. The child presented: centered corneal reflex, orthophoria to cover test, normal ocular motility, mild hyperopia without astigmatism in cycloplegia, no alterations of the retina and anterior segment, head tilt and restricted neck movements. Furthermore the patient showed intense suffering when an attempt was made to perform active contralateral neck rotation. Therefore we hypothesized that the child could be suffering from painful torticollis and neck pain. The parents reported no previous traumas to the head and neck and a sudden onset of AHP five days before. In accordance with the otolaryngologist and neurologist we required radiological insights. AAS was suspected by RX of the cervical spine. Three-dimensional computer tomography (CT) scan reconstruction and Nuclear Magnetic Resonance (NMR) revealed AAS (Fig. 1). It was decided to ask for an orthopedic evaluation.

**Fig. 2:**
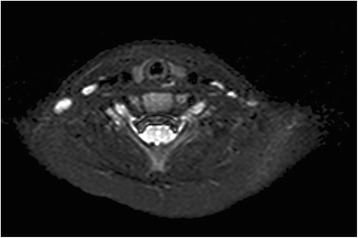
Axial NMR scan showing the recovery of AAS

Upon orthopedist’s suggestion, the patient started an antibiotic therapy (sulbactam + ampicillin 50 mg/kg/day iv), muscle relaxants, anti-inflammatory therapy for 2 weeks and cervical collar for 4 weeks. At the end of the therapeutic protocol, the AHP and painful torticollis resolved. Axial NMR examinations showed no abnormalities after 1 month (Fig. 2).

The diagnosis of GS is usually performed by orthopedists and/or otolaryngologists, but it might be difficult in very young patients (1–3 years old), due to low cooperation of patients and difficult interpretation of signs and symptoms. Especially in these cases, GS could be an exclusion diagnosis and an ophthalmological evaluation could be required. However, we believe that every child with AHP should be examined by an ophthalmologist.

Signs of GS should always be taken into account during ophthalmological examination: recent history of upper airway infections and/or head and neck surgeries associated to a new onset of sudden, painful AHP with normal ocular exam. In these cases blood samples could show altered parameters indicating infection, although this might not always be the case. Therefore, it is necessary to require quick the execution of radiological examinations (CT and/or NMR), which are essential to confirm the diagnosis.

Karkos et al. reported 96 cases with non-traumatic atlantoaxial rotary subluxation: 48 % due to infections and 40 % after ear, nose and throat surgery (adenotonsillectomy in 78 %). The infectious etiology includes: upper respiratory tract viral infection (83 %), retropharyngeal abscess (11 %), otitis media (4 %) and mumps (2 %) [16].

Grisel’s syndrome is an uncommon cause of AHP in children and it is often misdiagnosed. Different factors may lead to the atlanto-axial instability and to the subluxation of the atlanto-axial joint. The most important is the trasverse and alar ligaments inflammation generally associated with head and neck infections or surgical procedures [15, 17]. This could be the result of the direct connections between the periodontoid venous plexuses and pharyngo-vertebral veins that could lead peripharyngeal septic effusions to the atlanto-axial ligaments.

Other causes include larger head size, weaker cervical muscles, looser ligament and joints, shallower and more horizontally placed facet joints and existence of a lymphatic system with a greater number of retropharyngeal lymph nodes. These conditions are more prevalent in children, especially under 4 years of age and in Down syndrome patients [17–19].

The GS symptoms are neck pain and torcicollis resulting in limited neck rotation. The typical head position is 20° of homolateral tilt, 20° of heterolateral rotation, and slight flexion [11, 13]. It is mandatory to exclude other causes of torticollis, in particular posterior fossa and spinal cord tumors and congenital AHP [18]. Therefore the multidisciplinary approach with neurologist and orthopedic is recommended. The sudden onset of AHP and the age of the patients (5–12 years old) [7], could help to differentiate GS from congenital muscular torticollis. The latter in fact is more common in newborn patients [20].

Furthermore three clinical signs could be useful to distinguish AAS from other torcicollis. The first is the deviation of the axis of the spinous process in the same direction of head rotation. Otherwise in normal head rotation, the C2 process deviates to the heterolateral side. The second finding is the spasm of the homolateral sternocleidomastoid muscle to avoid neck pain [18, 21]. The third sign is the impossibility to turn the head beyond the midline in the opposite direction to the injury [13, 18, 21]. However 3D-CT reconstructions and NMR are very helpful in assessing the diagnosis.

To avoid severe neurological complications is mandatory an early diagnosis [17, 22, 23]. The correct management of that syndrome could lead a good prognosis. As said before, GS is usually caused by an infectious process, thus the first line medical treatment is characterized by antibiotic and anti-inflammatory therapy, muscle relaxants and cervical collar.

Deichmueller et al. reported that in 8 of their 12 patients it was found a complete remission after intravenous antibiotics and oral anti-inflammatory therapies. Only four patients required other intervention. Generally patients who do not respond to medical therapies have experienced torticollis for more than 2 weeks without any treatment [6, 24].

Finally, the surgery is recommended only in patients not responding to conservative treatment, who show an irreversible deformity [13, 21]. In the literature favorable results have been reported using surgical approach to reduce the AAS, even if there is no agreement on the best surgical procedure.

Neurological complications are uncommon, equal to 15 % of cases. These can range from radiculopathy to quadriplegia and death from medullary compression and acute respiratory failure [25]. These various complications depend on the severity of atlanto-axial joint subluxation.

## Conclusion

Interdisciplinary collaboration between ophthalmologists, pediatricians, pediatric surgeons, otorhinolaryngologists and neurologists is fundamental in establishing the etiology of AHP.

We underline the importance of an accurate orthoptic and ophthalmological examination to exclude an ocular disorder and suspect GS in patients complaining for a sudden onset of AHP associated to neck pain, recent history of upper airway infections and/or head and neck surgeries. Early diagnosis and treatment avoids surgery, tragic outcomes and ensures the success of a conservative approach.

### Consent

Written informed consent was obtained from parents of the patient (the patient is under 18 years old) for publication of this case report and any accompanying images. A copy of the written consent is available for review by the Editor of this journal.

## Abbreviation

AHP: Anomalous head posture
GS: Grisel’s syndrome
AAS: Atlanto-axial subluxation
CT: computer tomography
NMR: Nuclear Magnetic Resonance
